# COVID-19 and chronic kidney disease: a comprehensive review

**DOI:** 10.1590/2175-8239-JBN-2020-0203

**Published:** 2021-04-09

**Authors:** Inah Maria D. Pecly, Rafael B. Azevedo, Elizabeth S. Muxfeldt, Bruna G. Botelho, Gabriela G. Albuquerque, Pedro Henrique P. Diniz, Rodrigo Silva, Cibele I. S. Rodrigues

**Affiliations:** 1 Universidade Estácio de Sá, Curso de Medicina Rio de Janeiro, Campus Centro I - Presidente Vargas, Rio de Janeiro, RJ, Brasil.; 2 Universidade Federal do Rio de Janeiro, Hospital Universitário Clementino Fraga Filho, Rio de Janeiro, RJ, Brasil.; 3 Pontifícia Universidade Católica de São Paulo, Faculdade de Ciências Médicas e da Saúde, São Paulo, SP, Brasil.

**Keywords:** Renal Insufficiency, Chronic, Renal Dialysis, Peritoneal Dialysis, Mortality, Morbidity, Insuficiência Renal Crônica, Diálise Renal, Diálise Peritoneal, Mortalidade, Morbidade

## Abstract

Kidney impairment in hospitalized patients with SARS-CoV-2 infection is associated with increased in-hospital mortality and worse clinical evolution, raising concerns towards patients with chronic kidney disease (CKD). From a pathophysiological perspective, COVID-19 is characterized by an overproduction of inflammatory cytokines (IL-6, TNF-alpha), causing systemic inflammation and hypercoagulability, and multiple organ dysfunction syndrome. Emerging data postulate that CKD under conservative treatment or renal replacement therapy (RRT) is an important risk factor for disease severity and higher in-hospital mortality amongst patients with COVID-19. Regarding RAAS blockers therapy during the pandemic, the initial assumption of a potential increase and deleterious impact in infectivity, disease severity, and mortality was not evidenced in medical literature. Moreover, the challenge of implementing social distancing in patients requiring dialysis during the pandemic prompted national and international societies to publish recommendations regarding the adoption of safety measures to reduce transmission risk and optimize dialysis treatment during the COVID-19 pandemic. Current data convey that kidney transplant recipients are more vulnerable to more severe infection. Thus, we provide a comprehensive review of the clinical outcomes and prognosis of patients with CKD under conservative treatment and dialysis, and kidney transplant recipients and COVID-19 infection.

## Resumo

O comprometimento renal em pacientes hospitalizados com infecção por SARS-CoV-2 está associado ao aumento da mortalidade hospitalar e pior evolução clínica, levantando preocupações em relação a pacientes com doença renal crônica (DRC). De uma perspectiva fisiopatológica, a COVID-19 é caracterizada por uma superprodução de citocinas inflamatórias (IL-6, TNF-alfa), causando inflamação sistêmica e hipercoagulabilidade, e síndrome de disfunção de múltiplos órgãos. Dados emergentes postulam que a DRC sob tratamento conservador ou terapia renal substitutiva (TRS) é um fator de risco importante para a gravidade da doença e maior mortalidade hospitalar entre pacientes com COVID-19. Em relação à terapia com bloqueadores RAAS durante a pandemia, havia uma suposição inicial de que a classe pudesse causar um aumento potencial na infectividade, e impacto deletério na gravidade da doença e mortalidade, mas que não foi confirmada na literatura médica. Além disso, o desafio de implementar o distanciamento social em pacientes que necessitam de diálise durante a pandemia incentivou sociedades nacionais e internacionais a publicar recomendações sobre a adoção de medidas de segurança para reduzir o risco de transmissão e otimizar o tratamento de diálise durante a pandemia COVID-19. Os dados atuais mostram que os receptores de transplante renal são mais vulneráveis a infecções mais graves. Assim, fizemos uma revisão abrangente dos desfechos clínicos e prognóstico de pacientes com DRC sob tratamento conservador e diálise, e receptores de transplante renal e infecção por COVID-19.

## Introduction

In December 2019, cases of atypical pneumonia began to rise in the city of Wuhan, located in the providence of Hubei, China^1^. In March 2020, amid initiation of global spread, the World Health Organization (WHO) declared the outbreak a pandemic, caused by SARS-CoV-2, a new positive-strand RNA virus from the *coronoviridae* family, being from the same family of the viruses responsible for the severe acute respiratory syndrome (SARS) in 2002 and the middle east respiratory syndrome (MERS) in 2012^2^^-^ 4. In Brazil, until mid-September, the country surpassed 4,100,000 confirmed cases and 130,000 deaths due to the disease^2^^,^ 3. The etiological agent of COVID-19 is more infectious than SARS and MERS, with a basic number of reproductions (R0) ranging from 2-3.5^5^^-^ 7. Moreover, besides a high transmission rate, authors postulate that a crucial factor regarding the transmission of COVID-19 infection is the high level of SARS-CoV-2 present in the upper respiratory tract, even among pre-symptomatic patients, contributing to the global spread of the disease^5^^-^ 8.

From a pathophysiological perspective, COVID-19, especially in severe forms, is characterized by an overproduction of inflammatory cytokines due to cytokine storm triggered by viral infection, leading to systemic inflammation and a prothrombotic state^9^^,^ 10. Thus, besides lung involvement, other organ complications are observed in patients with SARS-CoV-2 infection such as kidney damage leading to acute kidney injury (AKI)^11^, raising concerns regarding the clinical outcomes and prognosis of patients with preexisting comorbidities such as chronic kidney disease (CKD), end-stage kidney disease (ESKD), and kidney transplant recipients under immunosuppression therapy.

**Figure 1:**
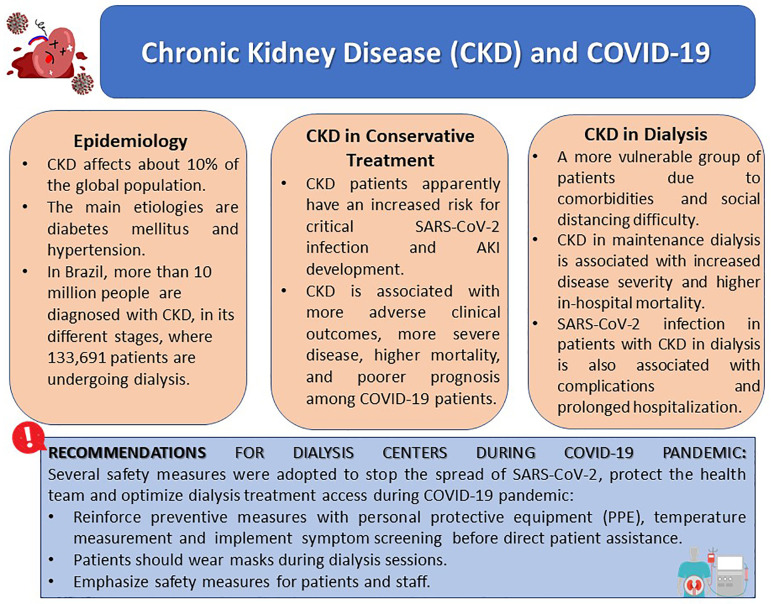
COVID-19 in patients with Chronic Kidney Disease. Brief summary of the key points regarding SARS-CoV-2 infection in patients with prior CKD undergoing conservative or dialytic therapy.

A meta-analysis including 73 studies evaluating the association between multi-organ dysfunction and COVID-19 development revealed that patients with CKD were more likely to develop severe SARS-CoV-2 infection (OR 1.84 [95%CI 1.47-2.30])^12^. Hence, besides disease severity, it is imperative to evaluate the clinical outcomes, prognosis, and mortality associated with COVID-19 infection in patients with history of CKD, CKD on maintenance dialysis, and kidney transplant recipients (Figure 1).

## Methodology

A thorough scoping review based on the PubMed electronic bibliographic database was performed between April and September 2020, using the following Mesh terms: "Renal" OR "Kidney", OR "Hemodialysis", OR "Peritoneal Dialysis", OR AND "Chronic Kidney Disease" AND "Kidney Transplant Recipients" AND "COVID-19", with adoption of the PICO strategy and classification of the level of evidence.

The guiding question to construct the review was: what is the latest scientific evidence regarding SARS-CoV-2 in patients with COVID-19 and chronic kidney disease? Articles diverging from the central theme were excluded from the review. After exclusion, 90 articles were selected and cited directly or via cross-reference in the present review.

## Integrated Discussion

### Patients with ckd under conservative treatment and covid-19

In 2017, CKD affected around 10.0% of the global population. In Brazil, more than 10 million presented the disease in its different stages, with 139,691 undergoing dialysis of which 93.2% in hemodialysis in 2019^13^^-^ 15. In addition to the high prevalence, CKD remarkably increases morbimortality and is associated with higher infection risk, mainly respiratory, and a more precise comprehension of the prognosis and the clinical evolution of CKD patients infected by COVID-19 is crucial^16^. In a study evaluating the early predictors of clinical outcomes of COVID-19 outbreak in Milan, Italy, the prevalence of CKD amongst hospitalized patients with COVID-19 was 11.8%^17^.

De Lusignan et al. in a recent cross-sectional study describing the risk factors for SARS-CoV-2 infection among 3,802 patients in the Oxford Royal College of General Practitioners Research and Surveillance Centre primary care network found that individuals with CKD were more likely to test positive for COVID-19 (68 [32.9%] of 207 with CKD vs. 519 [14.4%] of 3595 without CKD; OR 1.91 [CI95% 1.31-2.78])^18^.

The high incidence of kidney involvement observed in hospitalized patients with COVID-19 might be also due to the presence of previous chronic kidney impairment. Cheng et al., in a prospective analysis of 701 patients with COVID-19, demonstrated that in comparison with patients with normal serum creatinine (SCr), patients admitted with elevated SCr presented higher leukocyte count (9.5±8.0 vs. 7.2±7.4 x 10^9^/L, p=0.005), lower lymphocyte count (0.8±0.5 vs. 0.9±0.5 x 10^9^/L, p=0.015), lower platelet count (191±94 vs. 216±84 x 10^9^/L, p=0.014), prolonged partial thromboplastin time >42s (54.2 vs. 40.4%, p=0.029), higher D-dimer levels (>0.5mg/L) (89.8 vs. 75.3%, p=0.002), increased procalcitonin (≥0.5ng/mL) (29.3 vs. 6.9%, p<0.001), increased lactose dehydrogenase (LDH) (458±254 vs. 364±180, p=0.001), and the incidence of AKI was significantly higher in patients with elevated baseline SCr (11.9 vs. 4.0%, p=0.001). Furthermore, patients with COVID-19 and elevated baseline SCr presented higher prevalence of intensive care unit (ICU) admission (12.8 vs. 10.0%, p=0.382) and mechanical ventilation (21.8 vs. 12.8%, p=0.012). After univariate Cox regression analysis, elevated baseline SCr increased the risk of in-hospital death by almost three-fold (HR 2.99 [CI95% 2.00-4.47], p<0.001). CKD *per se* is associated with a proinflammatory state, inferring that patients with chronic kidney impairment and COVID-19 might evolve with a more pronounced cytokinetic storm, resulting in more severe systemic inflammation and hypercoagulability, being an important risk factor for acute kidney injury, severe illness, and mortality^19^.

The prospective analysis performed by Cheng et al. conjectures that previous chronic kidney impairment might have a negative impact on the clinical evolution and fatality risk of COVID-19^19^. Moreover, an analysis of the international Health Outcome Predictive Evaluation for COVID-19 registry (HOPE-COVID-19) evaluating the impact of renal function on admission and mortality in 758 patients with SARS-CoV-2 infection revealed a CKD prevalence of 8.5% amongst infected patients, and 30.0% had kidney dysfunction upon admission (eGFR <60mL/min/1.73m^2^)^20^. Patients were allocated into three groups according to the eGFR upon admission: absence of significant renal failure (eGFR >60mL/min/1.73m^2^), moderate renal failure (eGFR 30-60mL/min/1.73m^2^), and severe renal failure (eGFR <30mL/min/1.73m^2^). Patients with kidney dysfunction upon hospital admission presented a higher incidence of complications such as sepsis (11.9 vs. 26.4 vs. 40.8%, p<0.001) and respiratory failure (35.4 vs. 72.2 vs. 62.0%, p<0.001). Moreover, the incidence of AKI during admission was 19.7%, and patients with more severe kidney dysfunction upon admission were more susceptible for kidney function worsening during hospitalization (eGFR> 60mL/min/1.73m^2^ = 5.2% vs. eGFR 30-60mL/min/1.73m^2^= 31.8% vs. eGFR<30mL/min/1.73m^2^= 56.0%, p<0.001). Kaplan-Meier survival landmark analysis according to GFR demonstrated that the survival probability after 20 days was remarkably lower in patients with eGFR<30mL/min/1.73m^2^ (22.8%) and eGFR 30-60mL/min/1.73m^2^ (27.2%) compared to patients with absence of significant renal failure during hospital admission (71.7%). After Cox multivariate regression analysis, worse kidney function during hospital admission was an independent factor for in-hospital mortality as eGFR 30-60mL/min/1.73m^2^ increased in two-fold the risk of death (HR 2.205 [95%CI 1.573-3.091], p<0.001) and eGFR<30mL/min/1.73m^2^ increased almost five-fold the risk for in-hospital death amongst COVID-19 patients (HR 4.925 [95%CI 2.152-5.244], p<0.001)^20^.

Another prospective cohort study including 1,821 patients admitted to a University reference hospital in Spain revealed that 43.5% of patients with elevated SCr levels on hospital arrival had previous history of CKD and that the raw in-hospital mortality rate was higher in patients with increased SCr (32.4%), patients with previous CKD (41.1%), and patients who developed AKI during hospitalization (15.9%) compared to patients with normal SCr (5.8%). Additionally, the Kaplan-Meier analysis of cumulative incidence for in-hospital death revealed that patients with previous history of CKD and patients with elevated SCr levels on admission presented higher 20 day-mortality than patients with normal baseline creatinine. Elevated SCr on hospital admission (HR 4.07 [95%CI 3.07-5.39]) and previous history of CKD (HR 4.17 [95%CI 3.08-5.66]) were also associated with higher in-hospital death in the univariate Cox regression analysis. Thus, these studies accentuate that history of previous kidney impairment during hospital admission seems to be an independent risk factor for worse prognostic, urging that CKD history and kidney function must be screened during triage in patients with confirmed or suspected COVID-19^19^^-^ 21.

In an initial meta-analysis by Henry and Lippi published in March including four studies involving a total of 1,389 patients infected by SARS-CoV-2, the presence of CKD tripled the risk of patients developing severe disease [OR 3.03 (95%CI 1.09-8.47)]. Despite the low heterogeneity between the studies, none specifically evaluated and considered CKD as a pre-existing disease or obtained statistical significance, creating uncertainties about this association^22^. Nonetheless, the association between CKD and more severe COVID-19 was strengthened and clarified by subsequent studies.

Abrishami et al. in a single-center study evaluating the clinical and radiological characteristics of 43 adult CKD patients with confirmed COVID-19 in Iran, described that patients with CKD are vulnerable to a more severe form of COVID-19 and are predisposed to a higher mortality rate than the general population. The mean age of patients was 60.65±14.36 years and the most frequent CKD stage was IIIa (44.2%) and the least common was stage IV (4.7%), highlighting that amongst the total 43 CKD patients with COVID-19, 38 (88.4%) were discharged and 5 (11.6%) died on follow-up. The most prevalent symptoms were dyspnea (65.1%) and cough (60.5%). Laboratory evaluation revealed that leukopenia, leukocytosis, and thrombocytopenia were observed in 7 (16.3%), 4 (9.3%), and 12 (27.9%) patients, respectively. Moreover, LDH serum levels were significantly higher in CKD patients who died (740.2 ± 452.9 vs. 355 ± 127.5 IU/L). No significant laboratory alteration was observed across the CKD stages (p>0.05). Regarding CT scan findings, bilateral lung involvement was observed in 93.0% of the patients, the most common pattern of lung involvement was ground glass opacification (35.9%) and reticular pattern (16.3%), and the prevalence of pleural and pericardial effusion were 20.0 and 14.0%, respectively. Moreover, ground glass opacification was significantly higher in patients who died in comparison to survivors (60.0% vs. 31.5%). Regarding the analysis by CKD groups, the extent of lung involvement evaluated by total lung score significantly differ (p>0.05). On admission, 58.1% of CKD patients had severe COVID-19 and the mean duration of hospitalization was 11.65± 6.67 days, being more prolonged in patients with stage V CKD (15.4±6.4 days) and patients who died (16.6. ±8.38 days), despite lack of statistical significance (p>0.05)^23^. Thus, despite a high prevalence of severe disease and high mortality, higher CKD stage was not significantly associated to a worse prognosis^23^.

HOPE-COVID-19 investigators also demonstrated that from the 758 patients included in the study, patients with poorer kidney function (GFR 30-60mL/min/1.73m^2^ and GFR <30mL/min/1.73m^2^) on hospital admission had more adverse clinical manifestations and laboratory findings compared to patients with absence of significant renal failure (GFR >60mL/min/1.73m^2^)^19^. Shortness of breath (55.8 vs. 59.8 vs. 67.2%), tachypnea (19.0 vs. 28.1 vs. 39.9%) and, oxygen saturation on admission <92% (30.5 vs. 59.8 vs. 52.8%) were more frequent in patients with lower GFR during hospital admission. Furthermore, regarding laboratory profile, patients with poorer kidney function evolved with more significant D-dimer elevation (61.% vs. 79.% vs. 65.8%), and procalcitonin elevation (19.5 vs. 26.5 vs. 48.7%). More impaired kidney function during hospital admission was associated with a notably higher incidence of AKI (6.7 vs. 43.4 vs. 86.3%) and acute respiratory distress syndrome (ARDS) (35.4 vs. 72.2 vs. 62.0%). The prospective analysis from Spain corroborates these findings as patients with CKD presented increased inflammatory biomarker values such as CRP (113.7 vs. 65.6 mg/L, p=0.009) and ferritin (1132 vs 8721ng/mL, p=0.04), and altered coagulation markers as elevated D-dimer (>1.7mg/dL (%) (56.5 vs. 34.7%, p<0.001) and prolonged activated partial thromboplastin time (46.1 vs. 38.5 s, p<0.001) than non-CKD patients with COVID-19^20^^,^ 21.

A nationwide retrospective case-control study including 2,019,961 individuals evaluating the effect of underlying comorbidities on the severity of COVID-19 in Korea reported that CKD and ESKD were associated with severe COVID-19 (OR 2.052-2.178)^24^. Furthermore, a meta-analysis and systematic review including 34 studies also demonstrated that CKD (OR 3.02 [95%CI 2.23-4.08]) was associated with more severe and fatal outcomes among patients with COVID-19^25^. Fried et al. in an observational cohort study assessing clinical characteristics and outcomes of 11,271 patients with COVID-19 hospitalized in 245 hospitals across 38 different states in the United States revealed that CKD was associated with a higher need for mechanical ventilation (OR 1.22 [95%CI 1.05-1.43])^26^. Moreover, a cross-sectional study of 212,802 confirmed COVID-19 cases from Mexico demonstrated that comorbidities such as previous history of CKD increased the severity of COVID-19. The study found a correlation between CKD and a higher risk of hospitalization (OR 2.54 [95%CI 2.36--2.73]), ICU admission (OR 1.12 [95%CI 0.97-1.29]), and endotracheal intubation (OR 1.30 [1.15-1.48])^27^.

Besides more adverse clinical outcomes and heightened severity, CKD also seems to be associated with a higher mortality in patients with SARS-CoV-2 infection. Williamson et al. recently described the factors associated with COVID-19-related death using primary records of 17,278,392 adults pseudonymously linked to 10,926 COVID-19 related deaths with a secure health analytics platform from NHS England called OpenSAFELY^28^. The study emphasizes the significance of CKD as an important risk factor for COVID-19 mortality, as estimated hazard ratios from a multi-variable model associated CKD with eGFR 30-60 (HR 1.33 [95%CI 1.28-1.40]) and eGFR <30 (HR 2.52 [95%CI 2.33-2.72]) as a risk factor for mortality in patients with COVID-19^28^.

A retrospective observational cohort study evaluating the risk factors associated with mortality among 3,988 critically ill patients with laboratory-confirmed COVID-19 referred for ICU admission in the region of Lombardy in Italy revealed a high mortality in patients with CKD. Among the first 1,715 patients, the prevalence of CKD was 3.1% and of 52 patients with CKD admitted to the ICU, 41 died (78.8%) and 11 were discharged from ICU (21.2%). Regarding mortality in the hospital setting, 44 patients with CKD died (84.6%) and 7 were discharged from the hospital (13.5%). Analyzing the full cohort of 3,988 patients, 87 patients had previous history of CKD (2.2%) and 71 with CKD died (81.6%). After univariate analysis, CKD was associated with higher mortality (HR 2.78 [2.19-3.53], p<0.001) in patients with COVID-19 admitted to the ICU^29^.

An analysis of 3,391 patients positive for COVID-19 in the Mount Sinai hospital in New York demonstrated that without adjusting for age groups, patients with CKD had a higher risk of mortality (RR 2.51 [95%CI 1.82-3.47], p<0.001) and intubation (RR 2.05 [1.40-3.01], p<0.001). Moreover, amongst CKD patients, a significantly higher rate of death was observed in patients with atrial fibrillation (OR 2.13 [95%CI 1.03-4.43]), heart failure (OR 2.09 [1.16-3.77]), and ischemic heart disease (IHD) (OR 2.87 [1.04-3.36])^30^. Fang et al. in a meta-analysis and systematic review including 61 studies also associated CKD with higher mortality (RR 7.10 [3.14-16.02], p<0.001), increasing by seven-fold the risk of death in patients with SARS-CoV-2 infection^31^. Thus, CKD seems to be an important risk factor for disease severity and higher in-hospital mortality^27^^-^ 34.

The interaction between SARS-CoV-2 and the RAAS system raised concerns regarding the use of RAAS inhibitors during the COVID-19 pandemic, due to the possibility of enhanced virulence and infectivity, worsening the prognosis of these patients. Nonetheless, despite ACE-2 being later identified as a receptor for SARS-CoV-2 cell invasion, evidence initially suggesting that the use of RAAS blockers might increase the expression of ACE-2 in the heart and kidneys were not confirmed^35^.

In a retrospective study with 12,594 individuals who underwent COVID-19 tests, the use of RAAS inhibitors was not associated with a higher risk of contamination nor a worse evolution of the disease among patients infected by SARS-CoV-2^36^. In another case-control study, Mancia et al. evaluated the impact of RAAS inhibitors in the severity of the disease in 6,272 patients who tested positive for COVID-19, in comparison with the control group of the target population^37^. Analogously, no association between the use of these anti-hypertensive agents and a more severe evolution of COVID-19 was demonstrated^36^^,^ 37.

Correspondingly, a meta-analysis of the effects of RAAS blockers (ACE inhibitors and angiotensin 2 AT_1_ receptor blockers) in patients with COVID-19 compared 308 individuals using RAAS blockers and 1,172 individuals undergoing treatment with other antihypertensive drugs^38^. Severity of disease, risk of hospitalization, and death were the main outcomes of interest assessed. Patients with COVID-19 who were taking RAAS blockers had a lower risk of developing severe illness (44.0%), lower risk of death (62.0%), and reduced hospitalization (19.0%), although reduced hospitalization did not obtain statistical significance^38^^-^ 41.

Therefore, the initial assumption of a potential increase in infectivity and morbimortality in patients with CKD, hypertension, and/or heart failure undergoing treatment with RAAS blockers during the COVID-19 pandemic and the lack of robust scientific data evidencing a deleterious impact, prompted national and international societies to issue positions urging RAAS inhibitors maintenance in patients with formal indication. Additionally, a non-deleterious impact of RAAS blockers in SARS-CoV-2 clinical evolution has been proven by subsequent papers^37^^,^ 38 ^,^ 42 ^-^ 46.

The BRACE CORONA trial, the first multi-center randomized controlled study evaluating the safety of ACE inhibitors and ARBs on hospitalized patients with mild to moderate COVID-19 in 659 enrolled patients from 29 distinct sites in Brazil, revealed that among patients with COVID-19 infection undergoing chronic ACEi/ARB therapy, suspending ACEi/ARB did not improve the number of days alive and hospital discharge in 30 days (21.9 vs. 22.9, p=0.009), and a similar 30-day mortality rate was observed in COVID-19 patients who continued or suspended ACEi/ARB therapy (2.8 vs. 2.7%, p=0.95), highlighting that there is no clinical benefit from ACEi inhibitor/ARB treatment interruption in hospitalized patients with mild to moderate COVID-19^47^.

Studies are still required for a better comprehension of the peculiarities, clinical outcomes, and prognosis of non-dialytic CKD patients with COVID-19, elucidating questions about comorbidities as confounding factors and their immunological profile, to obtain the best possible outcomes for these patients. The main findings of studies involving individuals with CKD under conservative treatment are summarized in Table 1.

**Table 1:** Summary of the major studies regarding CKD under conservative treatment and COVID-19

Author	N	Design	Age (years)	Comorbidities	Major findings
Uribarri et al.	758	Cohort			1.Mortality risk:
					(Kaplan-Meier survival curve):
					-eGFR > 60mL/ min/1.73 m^2^ = 71.7%
					- eGFR 30-60 mL/ min/1.73 m^2^ = 27.2%
				HTN (48.9%)	- eGFR < 30mL/ min/1.73 m^2^ = 22.8%
			66.0	DLP (38.7%)	2. Risk factors on admission associated with in-hospital death (multivariate regression):
			±18.0	DM (21.9%)	- Age: (HR 1.034 [CI95%1.021-1.048]; p<0.001)
				CKD (8.5%)	- SatO_2_ <92.0%: (HR 3.310 [2.362-4.369]; p<0.001)
					- eGFR 30-60: (HR 2.205 [1.473-3.091]; p<0.001)
					- eGFR < 30 (HR 4.925 [2.152-5.244]; p<0.001)
Ji et al.	219,961	Retrospective		HTN (22.2%)	
			47.05	DM (14.2%)	1. Severe COVID-19: (multivariate analysis)
			±18.0	CAD (4.2%)	-CKD/ESKD: (OR 2.052-2.178)
				CKD (1.0%)	
Fried et al.	11,721	Retrospective			1. Severe COVID-19:
				HTN (46.7%)	-Mechanical ventilation
			> 60	DM (27.8 %)	(15.2% vs. 11.6%; p<0.001)
			(67.3%)	CVD (22.6 %)	(OR 1.22 [CI95% 1.05-1.43]).
				CKD (4.3%)	2.Mortality:
					-CKD (OR 1.66 [CI95% 1.45-1.91]).
Hernández-Galdamez et al.	212,802	Cross-sectional			1. Severe COVID-19:
					Hospitalization:
				HTN (20.12%)	i. CKD (OR 2.54).
			45.7	DM (16.44%)	ii. ICU admission:
			±16.3	CVD (2.35%)	CKD (OR 1.12).
				CKD (2.17%)	iii. Intubation:
					CKD (OR 1.30).
					2. Mortality:
					- CKD (OR 2.31).
Williamson et al.	17,278,392	Cohort	18-39 (34.2%)		
			40-49 (16.5%)		1.Mortality:
			50-59 (17.7%)	HTN (34.3%)	Kidney function:
			60-69 (13.8%)	CVD (6.8%)	i). eGFR 30-60 (HR 1.33 [1.28-1.40]).
			70-79 (11.2%)		ii) eGFR <30 (HR 2.52 [2.33-2.72]).
			>80 (6.5%)		
Grasselli et al.	3,988	Retrospective	-	HTN (41.2%)	1.Mortality:
				DM (12.9%)	- CKD (OR 2.78 [95%CI 2.19-3.53];p<0.001).
				CVD (13.4%)	

DM, diabetes mellitus; HTN, hypertension; CVD, cardiovascular disease; CAD, coronary artery disease; COPD, chronic obstructive pulmonary disease; CKD, chronic kidney disease; DLP, dyslipidemia; eGFR, estimated glomerular filtration rate; ESKD, end-stage kidney disease; ICU, intensive care unit.

### Patients with ckd in dialytic therapy and covid-19

Evidence concerning management of CKD patients on dialysis therapy infected with SARS-CoV-2 is still scarce. While these are high-risk patients due to the presence of comorbidities, especially hypertension, cardiopathies, left ventricular hypertrophy, diabetes mellitus, among others, it is not yet clear if dialysis therapy *per se* is associated with a worse prognosis in patients infected with SARS-CoV-2, although infections in general can decompensate underlying CKD^48^^-^ 52.

In a recent analysis of 37,852 patients in hemodialysis (HD) in Brazil, 1,291 patients were positive for SARS-CoV-2 infection and 357 patients died. Authors postulate that the incidence, mortality, and fatality rates in HD patients were 341/10,000 patients, 94/10,000 patients, and 27.7%, respectively, raising concerns regarding the vulnerability of this group amid the COVID-19 pandemic^53^.

Implementing social distancing in patients requiring dialysis is difficult due to need of frequent visits to dialysis clinics and direct contact with special care teams of clinics and hospitals, increasing the risk of COVID-19 dissemination and consequently the vulnerability of this group^50^^,^ 51.

Initial Chinese case report studies revealed that patients with CKD on dialysis, presented moderate clinical manifestations, with fever as the most prevalent symptom, whilst only a small group of CKD patients developed cough. A study carried out in Zhongnan hospital, Wuhan, described diarrhea as the most frequent manifestation in these patients^52^. Another study reported gastrointestinal manifestation as the most important initial complaint among patients on dialysis treatment, and due to the atypical symptomatology, the authors emphasized the importance of individualized patient approach to optimize COVID-19 diagnostic accuracy in this peculiar group with uncertain prognosis^52^^-^ 54.

Authors initially hypothesized that the presence of atypical clinical manifestations and a possible less severe evolution of COVID-19 in CKD patients undergoing dialysis was due to the immunodepression status of these patients, inferring that this group of patients might not develop the usual severe immune dysregulation and consequent cytokinetic storm of critical SARS-CoV-2 infection. This could be due to these patients evolving with more significant lymphopenia and lower serum cytokines when compared to infected patients without history of kidney disease^55^. Nevertheless, recent retrospective and observational studies with more robust epidemiological and clinical data of COVID-19 patients undergoing dialysis demonstrated an increased risk for adverse clinical outcomes and higher mortality in this group of patients.

Xiong et al., in a retrospective multicenter study evaluating the clinical characteristics of 131 patients undergoing hemodialysis with SARS-CoV-2 infection, revealed that the most common symptoms were fever (51.9%), fatigue (45.0%), cough (37.4%), sputum production (29.0%), and dyspnea (26%). Furthermore, 40 (30.5%) patients evolved with acute organ injury and dysfunction, including 24 (28.2%) with cardiac injury, 16 (15.5%) with liver dysfunction, 16 (13.8%) with ARDS, and 9 (9.6%) with cerebrovascular event. Regarding radiologic findings, the most common abnormalities revealed in CT scans were ground-glass or patchy opacities (82.1%) with bilateral lung involvement (86.7%), but foci of consolidation was uncommon (4.3%). Laboratory data revealed that the median levels of hemoglobin and lymphocytes were 105 x 10^9^ cells/L (IQR 91.0-118.0) and 0.7 x 10^9^ cells/L (IQR 0.5-1.1), respectively, and the majority of the patients had normal white cell and platelet counts^56^.

ESKD on chronic hemodialysis is also associated with higher short-term mortality, worse clinical evolution, and increased severity. A retrospective cohort study with 114 hospitalized patients on chronic hemodialysis with COVID-19 in New York demonstrated that 13.0% required ICU admission, 17.0% required mechanical ventilation, and in-hospital death occurred in 28.0% of these patients, being 87.0% of those who required ICU care^57^. Likewise, Valeri et al. retrospectively analyzing the clinical presentation and outcomes of 59 hospitalized patients with ESKD and COVID-19 revealed that 18 patients (31%) died in a median of 6 days after hospital admission, including 75.0% of patients who required mechanical ventilation. Moreover, patients who died presented higher initial median values of white blood cell count (7.5 vs. 5.7 x 1000/µL; p=0.04), lactate dehydrogenase (507 vs. 312 U/L; p=0.04), and C-reactive protein (CRP) (163 vs. 30.3 mg/L; p=0.01) in comparison to survivors^58^.

Tortonese et al. in a retrospective cohort study describing the demographics and clinical course of 44 patients on maintenance dialysis with COVID-19 in the Paris region also showed a correlation with worse outcomes and higher SARS-CoV-2 infection severity. The main coexisting comorbidities were hypertension (97.7%), dyslipidemia (59.1%), diabetes mellitus (50.0%), and obesity (34.1%), whilst the most prevalent symptoms were fever and chills (79.5%) and cough and shortness of breath (29.5%). Diarrhea, a frequent symptom in preliminary case reports, was present in 13.6% of patients^59^.

Laboratory evaluation revealed that most dialyzed ESKD patients with COVID-19 during hospitalization presented anemia (77.3%), hyperfibrinogenemia (77.3%), hyperferritinemia (70.5%), increased D-dimer levels (56.8%), lymphopenia (54.5%), and increased CRP levels (52.3%), depicting a more profound inflammatory and thrombotic profile. Moreover, aggravation of hematological and inflammatory markers was more remarkable in patients requiring oxygen therapy. Chest computed tomography scan performed in all 41 patients demonstrated a high prevalence of bilateral ground-glass opacities with or without consolidations (80.5%) and severe radiological findings were present in 31.7% of the cases^59^.

The retrospective analysis also demonstrated that COVID-19 in dialyzed patients was associated with a higher mortality rate, complications, and prolonged hospitalization. The median duration of hospitalization was 12 days (IQR 7-18) and the median length of stay in ICU was 10 days (IQR 7-21). Concerning severe adverse events, 27.3% of ESKD dialytic patients required mechanical ventilation, 27.3% evolved with ARDS, and 22.7%, with hemodynamic instability. In comparison with non-dialyzed patients, ESKD dialyzed patients presented higher mortality (27.3 vs. 12.9%, p=0.006), increased need for intensive care (34.1 vs. 22.7%, p=0.04) and remarkably higher in-ICU mortality (60.0 vs. 20.7%, p=0.002). After univariate Cox survival analysis, ARDS (HR 4.44 [95%CI 1.40-14.03], p=0.01), neutrophil count ≥10g/L (HR 4.49 [1.34-14.93], p=0.01), thrombocytopenia (HR 6.06 [1.64-22.49], p=0.003), metabolic acidosis (HR 11.18 [1.43-87.51], p=0.02), LDH levels ≥ 2 times the upper normal limit (HR 3.99 [1.26-12.63], p=0.016), blood CRP level ≥ 175mg/L (HR 13.06 [1.68-101.41], p<0.001), and D-dimer level > 4000 U/I (HR 4.44 [1.11-11.03], p=0.03) were associated with higher risk of death, being potential prognostic factors for mortality in hospitalized ESKD dialytic patients with COVID-19^59^.

A report from the Brescia renal COVID task force on the clinical characteristics and short-term outcomes of hemodialysis patients with SARS-CoV-2 infection also revealed a significant association with disease severity and in-hospital mortality. From a total of 94 patients, 57 (60.0%) required hospitalization after a median time from symptom onset of 4 days (IQR, 1-7) and from positive RT-PCR test results of 4 days (IQR, 1-3). Furthermore, 45 patients (79.0%) developed ARDS and 24 patients (42.0%) died after a median of 9 days (IQR, 7-10) from symptom onset. Among patients who died, the most frequent cause of death was respiratory failure secondary to ARDS (63.05%). Among survivors, 11 patients (19.0%) were discharged after a median of 8 days from admission (IQR, 6.5-13) and 15 days (IQR, 12.5-17.5) from onset of symptoms. After univariate logistic regression analysis, heart failure (OR 6.22 [CI95% 1.85-28.6]; p=0.007), ischemic heart disease (OR 5.61 [1.65-25.9]; p=0.01), fever at disease diagnosis (OR 18.2 [5.6-82.44]; p= 0.000013), shortness of breath at diagnosis (OR 18.17 [4.8-119.5]; *p* = 0.0002), myalgia or fatigue at diagnosis (OR 5.6 [1.65-25.9]; *p* = 0.01), infiltrates at the baseline chest X-ray (OR 4.4 [1.67-13]; *p* = 0.004), higher aspartate aminotransferase levels (OR 2.81 [1.08-7.6]; *p*= 0.04), and higher C-reactive protein levels (OR 4.68 [1.83-12.7]; *p* = 0.002) were associated with higher chance of developing ARDS during hospitalization. Additionally, ischemic heart disease (OR 3.11 [1.02-9.6]; p= 0.05), fever at disease diagnosis (OR 18.7 [3.62-343]; p = 0.005), cough at disease diagnosis (OR 3.5 [1.28-9.7]; *p*= 0.01), shortness of breath at disease diagnosis (OR 5.3 [2-15]; *p*= 0.001), and higher C-reactive protein level at disease diagnosis (OR 6.0 [2.1-19]; *p* = 0.001) were associated with higher mortality among hospitalized patients^60^.

Wang et al. in a retrospective single-center case series study in Zhongnan Hospital of Wuhan University evaluated the clinical outcomes of maintenance hemodialysis patients with COVID-19 and the impact of proactive chest CT scans. From 202 HD patients, 7 (3.5%) were diagnosed with SARS-CoV-2 infection, being 5 patients by RT-PCR and 2 patients diagnosed by RT-PCR as a result of screening 197 asymptomatic HD patients by chest CT scan. Regarding chest CT findings, 13 patients presented ground-glass opacity, but only 2 patients (15.0%) were confirmed to have COVID-19 by RT-PCR. Among the 7 patients with confirmed infection, all of them presented bilateral lung involvement. Lymphocytopenia (86%), elevated LDH (75%), elevated D-dimer (83%), elevated CRP (100%), and elevated procalcitonin (100%) were the most prevalent laboratory findings in infected HD patients. Moreover, 4 patients (57.0%) received oxygen therapy, 1 patient received noninvasive and invasive mechanical ventilation (14.0%), 1 patient developed ARDS (14.0%), and 3 patients died^61^. Additionally, another retrospective analysis of 31 hemodialysis patients with COVID-19 revealed an association with more severe illness and more adverse outcomes as 58.1% of patients presented organ dysfunction including ARDS (25.8%), acute heart failure (22.6%), and septic shock (16.1%)^62^. Besides worse clinical outcomes, a retrospective analysis of 14 consecutive patients on HD or with advanced CKD who initiated HD after COVID-19 diagnosis in South Korea demonstrated a prolonged median length of hospital and ICU stay in these patients, being 22.0 days and 6.0 days, respectively^63^.

The clinical outcomes of patients requiring chronic peritoneal dialysis (PD) associated with SARS-CoV-2 infection is also a concern for nephrologists. Sachdeva et al. in a case series study including 419 hospitalized patients with ESKD, 11 patients were on chronic PD (2.6%). Regarding clinical manifestations, the most prevalent symptoms were fever (64.0%), diarrhea (55.0%), shortness of breath (45.0%), cough (45.0%), and myalgias (36.0%). Majority of the patients presented bilateral opacities (82.0%) during initial chest imaging. Moreover, 3 patients (27.0%) were admitted to the ICU requiring mechanical ventilation. The length of hospital stay ranged from 2 to 23 days with a median of 9 days. Two patients died (18.0%) and 9 were discharged from the hospital (82.0%). Further studies with longer follow-up and a larger population are required for a more precise analysis concerning the clinical outcomes of chronic PD patients with COVID-19^64^.

Hence, SARS-CoV-2 infection in ESKD patients on maintenance dialysis seems to be associated with worse clinical outcomes, more profound inflammatory and thrombotic profile, more severe radiological findings, prolonged hospitalization, and higher fatality rate^57^^-^ 61 ^,^ 63.

Considering the hazardous context of SARS-CoV-2 infection, in order to attenuate the spread of the virus in CKD patients undergoing dialysis, a series of safety measures were adopted by hemodialysis centers and clinics to efficiently operate throughout the pandemic (Table 2)^65^^-^ 72. Nonetheless, Corbett et al. in a cohort study evaluating the epidemiology of COVID-19 in dialysis centers in the United Kingdom revealed that COVID-19 caused an abrupt epidemic in patients and healthcare workers. From the cohort of 1,530 patients with established kidney failure treated with dialysis in satellite units, 300 patients (19.6%) developed COVID-19^73^. In contrast, a study analyzing the incidence, clinical outcomes, and risk factors for mortality of COVID-19 in the French national cohort of dialysis patients demonstrated that the prevalence of COVID-19 varied from less than 1.0 to 10.0%^74^. Nevertheless, among 1,621 infected patients, 344 died (20.0%) and 9.0% were admitted to the ICU, highlighting that the mortality of ICU patients was higher compared to patients that did not require intensive care (35.0 vs. 15.5%). Risk factors for infection in dialysis patients were male sex (OR 1.2 [95%CI 1.1-1.4]), diabetes (OR 1.3 [1.1-1.4]), patients in need of assistance for transfer (OR 1.5 [1.3-1.8]), and patients treated in a self-care unit (OR 1.3 [1.0-1.6]). Moreover, at-home dialysis was associated with a lower SARS-CoV-2 infection probability (OR 0.6 [0.4-0.8])^74^. Despite lower incidence of SARS-CoV-2 infection compared with data from Corbett et al., patients on maintenance dialysis with COVID-19 presented high mortality, being imperative to reinforce health team protection and feasible logistics to secure patient safety and access to this indispensable treatment during this critical period^65^^,^ 70 ^-^ 74. High-risk of SARS-CoV-2 transmission, increased rates of hospitalization, and heightened morbimortality associated with ESKD patients on hemodialysis increased the support for home-based dialysis during the COVID-19 pandemic, particularly for PD modality^75^. The main findings of studies involving individuals with CKD under dialysis treatment are summarized in Table 3.

**Table 2:** Safety measures for dialysis centers during COVID-19 pandemic

Group	Main recommendations
**Hemodialysis patients**	**1. Education**
	✓ Patients should call dialysis clinics beforehand, optimizing specific individualized arrival logistics mitigating COVID-19 risk.
	✓ Patients should inform healthcare team of the presence of suspected COVID-19 symptoms before arrival.
	✓ Patients should be instructed on the proper use of PPE.
	✓ Patients should be instructed to safely self-isolate.
	**2. Screening**
	✓ Temperature screening for all patients upon arrival in dialysis clinics, being mandatory before and after sessions.
	✓ All patients should perform hand hygiene upon arrival in dialysis clinics.
	✓ All patients should wear personal protective equipment at all times during dialysis sessions.
	✓ Single use dialyzers of confirmed and/or suspected patients should be disposed.
	✓ All symptomatic dialytic patients must undergo rt-PCR screening test for COVID-19.
	✓ Symptomatic patients must be kept in isolation during dialysis sessions (6 ft of separation).
	✓ Patients with signs of critical infection must be immediately referred to a hospital.
**Healthcare team**	**3. Education**
	✓ PPE training for appropriate use.
	✓ PPE use at all times (isolation gown, gloves, mask, and eye protection).
	✓ Be vigilant towards COVID-19 symptoms.
	✓ Implementation of disinfection routine of all dialysis stations.
	✓ Emphasize and enhance dialytic patient’s knowledge regarding SARS-CoV-2 risks and infectivity.
	✓ Staying home if symptomatic.
	**4. Screening**
	✓ Body temperature measurement and symptom triage before contacting and assisting patients.
	✓ Symptomatic employees must be isolated and submitted to specific protocol.
	✓ All symptomatic healthcare workers should undergo rt-PCR screening test before patient assistance.

PPE: personal protective equipment.

**Table 3:** Summary of the major studies regarding CKD under dialysis and COVID-19

Author	N	Design	Age (years)	Comorbidities	Major findings
Xiong et al.	7,154	Retrospective			1. Clinical Manifestations:
				CVD (68.7%)	-Fever (51.9%), fatigue (45.0%), cough (37.4%), sputum (29.0%), and dyspnea (26.0%).
			63.1	DM (22.9%)	2. Clinical Evolution:
			(13.4)	COPD (3.8%)	-Acute organ dysfunction (30.5%),
				Cancer (1.5%)	- Cardiac injury (28.2%),
					- Liver dysfunction (15.5%),
					- ARDS (13.8%).
Fisher et al.	114	Cohort			1. Severe COVID-19:
				HTN (90.0%)	-ICU admission (13.0%).
			64.5	DM (67.0%)	-Mechanical ventilation (17.0%).
			(55.0-73.0)	CVD (55.0%)	2. Mortality:
				Cancer (12.0%)	-In-hospital death (28.0%):
					-ICU (87.0%).
					- General floor (19.0%).
Valeri et al.	59	Retrospective			1.Severe COVID-19:
					- Mechanical ventilation (14.0%).
				HTN (98.0%)	2. Mortality:
			63.0	DM (69.0%)	- In-hospital death: (31.0%).
			(56-70)	CAD (46.0%)	- Laboratory profile of patients who died compared to survivors:
				PD (17.0%)	- WBC (507 vs. 312 U/L; p=0.04).
					- CRP (163.0 vs. 80.3 mg/L; p=0.01).
					- LDH (507 vs. 312 U/L; p=0.04).
Tortonese et al.	44	Retrospective			1. Mortality
				HTN (97.7%)	Dialyzed x non-dialyzed:
			61.0	DM (50.0%)	1.1 Fatality Rate:
			(51.5-72.5)	DLP (59.1%)	-Patients requiring oxygen therapy: (36.4%); ICU patients: (60.0%); Non-dialyzed patients: (12.9%); Dialyzed patients: (27.3%).
				Obesity (34.1%)	1.2 Risk factors for mortality (Multivariate Cox analysis):
					- Cough (HR 5.18); thrombopenia ≤120g/L (HR 10.22); LDH ≥2N (HR 5.97); CRP ≥175mg/L (HR 19.53).
Alberici et al.	94	Retrospective			1. Risk factors for ARDS:
				HTN (93.0%)	-History of IHD (OR 7.5); fever at diagnosis (OR 17.0); Dyspnea at disease onset (OR 20).
			72.0	DM (43.0%)	2. Risk factors for mortality:
			(62.0-79.0)	CAD (17.0%)	- Fever (OR 18.7); cough (OR 4.0); Increased CRP (OR 5.6).
				Cancer (12.0%)	
Cécile et al.	1,621	Cohort			1. Severe COVID-19:
				DM (50.8%)	-ICU admission (9.0%).
			71.9	CAD (27.2%)	-Mechanical ventilation (51.0%).
			(60.8-81.0)	COPD (15.5%)	2. Mortality:
				Cancer (9.3%)	-Outpatients (8.5%):
					-Hospitalized (22.4%).
					- ICU (34.0% vs. 15.5%).

DM: diabetes mellitus; HTN: hypertension; CVD: cardiovascular disease; CAD: coronary artery disease; COPD: chronic obstructive pulmonary disease; PD: pulmonary disease; DLP: dyslipidemia; IHD: ischemic heart disease; ICU: intensive care unit.

### Kidney transplant recipients and covid-19

Evidence on the management and prognosis of kidney transplant recipients with COVID-19 is limited to case reports. In the vast majority of cases, the withdrawal or reduction of immunosuppressive therapy and the maintenance or introduction of corticosteroids were advocated, due to their immunomodulatory, anti-inflammatory, and vascular properties, which provide immunological protection to the renal allograft. However, while the ideal time for the reintroduction of immunosuppressive agents is quite uncertain, a prolonged reduction in immunosuppression increases the risk of graft rejection^76^^-^ 84.

Moreover, a significant part of preliminary reports show that kidney transplant recipients with COVID-19 have typical clinical symptoms, with fever and cough being quite recurrent^76^^,^ 78 ^,^ 84. There are also reports that, in addition to fever and cough, patients presented diarrhea and viral conjunctivitis^77^^,^ 85. Another important aspect observed in early case reports concerns radiographic alterations with unilateral or bilateral infiltrates during admission of these patients, of which most require ventilatory support, given the rapid decompensation observed among patients who develop ARDS^77^^-^ 84. Pulmonary complications, infectious or otherwise, are known to be an important cause of morbidity in patients undergoing immunosuppression^86^.^( )^It is also important to note that almost all patients in these reports presented comorbidities such as hypertension, diabetes, cancer, obesity, chronic respiratory diseases, and cardiovascular diseases, and some described the development of acute kidney injuries after hospital admission.

Devresse et al. described the clinical outcomes and mortality in a single center case series of 22 cases of COVID-19 in a cohort of 1,200 kidney transplant recipients in Belgium. From a total of 22 patients, 18 (82.0%) required hospitalization, and chest CT scan during admission performed in 15 patients revealed mild involvement in 3 patients (20.0%), moderate involvement in 8 patients (53.0%), severe involvement in 2 patients (13.0%), extensive involvement in 1 patient (7.0%), and critical involvement in 1 patient (7.0%). During hospital admission, the median baseline GFR was 45 (15-95) mL/min/1.73m^2^, median CRP was 56 (1.5-314) mg/L, and median lymphocyte count was 730 (50-1440)/µL. Moreover, 11 patients required supplemental oxygen therapy and 2 were admitted to the ICU requiring mechanical ventilation. Despite a small number of patients and a short follow-up period, after a period of 18 days, 13 (72.0%) of the 18 patients who required hospitalization were discharged from the hospital after a median of 10 days, however 3 (17.0%) patients were still hospitalized and 2 patients died (11.1%)^87^.

In another case series study with 12 patients evaluating the clinical course, imaging features, and clinical outcomes of COVID-19 infection in kidney transplant recipients, the most common symptoms were fever (75.0%), cough (75.0%), and dyspnea (41.7%), and only 1 patient had gastrointestinal symptoms. Leukopenia was observed in 4 patients (33.3%), leukocytosis in 1 patient (8.3%), CRP was elevated in 10 patients (83.3%), and creatine phosphokinase was elevated in five patients (55.0%). During hospital admission, mean BUN was 82.9±55.2 mg/dL and creatinine was 2.30±1.09 mg/dL. Initial CT scan on hospital admission revealed bilateral lung involvement in eight patients and unilateral involvement in four patients, the lower lobes were compromised in 11 patients, and a combination of consolidation and ground glass opacities (GGO) was the most prevalent pattern on the chest CT scan (75.05%). The authors postulate that interlobular septal thickening, multilobular patterns, consolidative lesions, and a high score for lung involvement were more prevalent among patients with more adverse outcomes and ARDS. Regarding clinical outcomes, 10 patients were admitted to the ICU, 9 were intubated, and 8 died of severe COVID-19 pneumonia and ARDS. The median length of hospital stay was 15 days (IQR 8.0-1.5) being longer in patients who died (18.0 days, IQR 12.3-21.5)^88^.

A prospective study assessing the clinical outcomes and the incidence of SARS-CoV-2 infection among 1,216 kidney transplant recipients revealed that patients with kidney transplant have a high risk of severe COVID-19. The most frequent symptoms were fever (77.0%) and cough (58.0%), and 60 patients (91.0%) required hospitalization. Furthermore, 15 patients (22.0%) required mechanical ventilation being transferred to the ICU. Notably, dyspnea was the most frequent symptom in patients admitted to the ICU, being observed in 12 of 15 (80.0%) patients in the invasive mechanical ventilation group compared with 27.0% in the non-invasive group. Also, the majority of patients requiring invasive mechanical ventilation had bilateral and multifocal lung opacities on chest x-ray or CT scan. The mortality rate related to COVID-19 disease in the cohort of kidney transplant population was 1.0%, nonetheless 16 of 66 (24.05%) kidney transplant recipients positive for COVID-19 died. After univariate logistic regression analysis, non-white ethnicity (OR 2.17 [95%CI 1.23-3.78], *p*=0.007), obesity (OR 2.19 [1.19-4.05, *p*=0.01), asthma and chronic pulmonary disease (OR 3.09 [1.49-6.41], *p*=0.002), and diabetes (OR 3.33 [1.92 to 5.77], *p*<0.001) were independently associated with COVID-19 in kidney transplant recipients^89^. Caillard et al. in a registry-based observational study including 279 transplant recipient patients with COVID-19 in France demonstrated a high 30-day mortality rate among this patient population (22.8%). Moreover, multivariable analysis identified age >60 years, cardiovascular disease, and dyspnea as independent risk factors for mortality in hospitalized patients^90^.

Studies are controversial due to their heterogeneity and a large number of confounders that influence the outcome of each case, such as the age of patients, time of transplantation, medications, and comorbidities. The future challenge is to identify the main clinical markers of poor prognosis in patients with kidney transplant, with additional studies with longer follow-up periods and more robust populations of immunosuppressed kidney transplant recipients. Table 4 summarizes the main findings of studies involving kidney transplant recipients.

**Table 4:** Summary of the major studies regarding kidney transplant recipients and COVID-19

Author	N	Design	Age (years)	Comorbidities	Major findings
Devresse et al.	22	Cohort			1. Clinical manifestations: .
				HTN (78.0%)	-Fever (78.0%), cough (67.0%), dyspnea (39.0%), digestive symptoms (28.0%), neurologic symptoms (16.0%)
			57.0	DM (22.0%)	2. Radiological presentation on CT:
			(41.0-73.0)	CVD (22.0%)	-Mild (20.0%), moderate (53.0%), severe (13.0%), extensive (7.0%), critical (7.0%).
				Obesity (22.0%)	
Abrishami et al.	12	Case series		0 (14.0%)	1. Clinical manifestations:
			66.0	1 (22.0%)	
			(57.0-76.0)	2 (25.0%)	-Fever (75.0%), cough (12.0%), dyspnea (41.7%).
				3 (13.0%)	2. Radiological presentation on CT:
				>4 (21.0%)	- Bilateral involvement (66.7%), GGO (100.0%), consolidation (75.0%), interlobular septal thickening (41.7%).
Elias et al.	1216	Prospective	56.4 ±13.4	DM (16.0%)	1. Factors associated with COVID-19 in patients with KT (multivariate analysis):
					- Non-White ethnicity (OR 2.17 [CI95% 1.23-3.78]; p=0.007), obesity (OR 2.19 [CI95% 1.19-4.05];p=0.01), asthma and COPD (OR 3.09 [CI95% 1.49-6.41; p=0.002), diabetes (OR 3.33 [CI95% 1.92-5.77];p<0.001).
Caillard et al	279	Observational			1.Clinical manifestations:
					-Symptoms: Fever (80.0%), cough (63.6%), diarrhea (43.5%), dyspnea (40.3%), and anosmia (14.1%).
					2.Laboratory profile:
					-CRP, mg/L (62 [27-144]); procalcitonin, ng/mL (0.20 [0.14-0.48]); lymphocyte count, x10^9^ (0.66 [0.40-0.96]); platelet count, x10^9^/L (178 [145-238); thrombocytopenia, <150 x10^9^/L (54 [29%]); creatinine, µmol/L (176 [131-244]).
					3.Radiographic profile:
				HTN (90.1%)	-Lung infiltrates on chest CT were detected in 87.0% of patients.
			61.6	DM (41.3%)	4.Clinical outcomes:
			(50.8-69.0)	CVD (36.2%)	-Complications: Acute kidney injury (43.6%), bacterial coinfection (23.5%), renal replacement therapy (11.1%), viral coinfection (2.1%), fungal coinfection (2.5%).
				Cancer (15.5%)	5.Severe COVID-19:
					-Oxygen therapy (72.4%), mechanical ventilation (29.6%), vasopressor support (11.1%).
					-ICU (36.0%); median interval between hospitalization and ICU admission was 4 days [1-25 days].
					Risk factors: >60yr (HR 1.63), BMI>25 kg/m^2^ (HR 1.80), diabetes (HR 1.73), dyspnea (HR 2.28), fever (HR 1.77), procalcitonin >0.2 (HR 3.19), SatO_2_ <95.0% (HR 2.47).
					6.Mortality: -30-day mortality rate: 22.8%
					Risk factors: age >60yr (HR 3.81), History of CVD (HR 2.04), dyspnea on hospital admission (HR 2.35).

DM, diabetes mellitus; HTN, hypertension; CVD, cardiovascular disease; BMI, body mass index; COPD, chronic obstructive pulmonary disease; ICU: intensive care unit.

## Conclusion

CKD under conservative treatment or maintenance dialysis seems to be associated with more adverse clinical outcomes, more severe disease, higher mortality, and poorer prognosis in patients with COVID-19 infection. Further studies are still required to elucidate the prognosis and clinical evolution of transplant kidney recipients. History of CKD must be taken into consideration during risk stratification of patients with confirmed or suspected COVID-19. Early detection of kidney abnormalities, optimal hemodynamic support when indicated, and avoiding nephrotoxic drugs with a risk-benefit judgement are essential steps to ensure a better evolution of these patients during hospitalization.
